# 

**Published:** 1874-02

**Authors:** 


### ACKNOWLEDGMENTS.

Atlanta Daily Herald; Charleston Medical Journal; Indiana Journal of Medicine; The Clinic; Medical and Surgical Reporter; Galaxy; Medical Examiner; London Lancet; Popular Science Monthly; Medical Record; American Journal Medical Sciences; Pacific Medical and Surgical Journal; Medical News and Library; Half Yearly Compendium Medical Sciences; Herald of Health; Western Lancet; Newspaper Reporter; Chicago Medical Journal; Cincinnati Lancet and Observer; North Western Medical and Surgical Journal; Methodist Advocate; Dunglison's Medical Dictionary; Griffith's Universal Formulary; Bellamy's Guide to Surgical Anatomy; Barnes on Diseases of Women; Leishman's System of Midwifery; Transactions of Maine Medical Association; Sarcomatous Growths of the Uterus, by Dr. Wm. F. Jenks, Surgeon to Pennsylvania Hospital for Women; Relations of Colorado to Pulmonary Consumption; The Larynx the Source of Vowel Sounds, by Thomas Bryan Gunning, New York.

